# Stability and robustness of asymptotic autocatalytic systems

**DOI:** 10.1038/s41598-020-72580-9

**Published:** 2020-09-23

**Authors:** Sohyoun Yun-Cárcamo, Sebastián Carrasco, José Rogan, Paulina Correa-Burrows, Juan Alejandro Valdivia

**Affiliations:** 1 Departamento de Física, Facultad de Ciencias, Universidad de Chile, Casilla 653, 7800024 Santiago, Chile; 2 Instituto de Nutrición y Tecnología de los Alimentos, Universidad de Chile, 7840390 Santiago, Chile

**Keywords:** Biocatalysis, Biophysical chemistry, Computational biophysics

## Abstract

Here, we address the consequences of the extension in the space of a simple model of a system that is closed to efficient causation: the (M,R)-system model. To do so, we use a diffusion term to describe the collective motion of the nutrients’ concentration across the compartmentalized space that defines the organism. We show that the non-trivial stable steady state remains despite such generalization, as long as the system is small enough to deal with the transport of the precursors to feed the entire protocell and dispose of a sufficient concentration of it in its surroundings. Such consideration explains the emergence of a bifurcation with two parameters that we characterize. Finally, we show that the robustness of the system under catastrophic losses of catalysts also remains, preserving the original’s model character.

## Introduction

“What makes life possible?” and “What are the ingredients of life?” remain some of the most exciting open questions guiding today’s research about what makes the matter a living thing^1–4^. In the long term, we could dream, and expect, that computer-based simulations of living systems may help us explain how a combination of atoms can result in a highly complex living entity such as a bacterium, a plant, an animal, or even a human being. Among other proposals, NASA’s definition may provide a clue for some of the ingredients of life that such simulations may have to provide: “life is a self-sustaining chemical system capable of Darwinian evolution”^5^. Although, it has been theorized that there might exist life beyond Darwinian evolution^2^.

Understanding autocatalysis as a metabolic closure process could be relevant for the comprehension of the origin and maintenance of life^6–10^. Robert Rosen’s *Metabolism-Replacement systems*^6^ or (M,R)-systems, provide a straightforward example of processes containing some of the life ingredients, such as the requirement of a self-sustaining chemical system through autocatalysis, which is a common requirement to most definitions of life^11^. However, the (M,R)-system seems to lack the Darwinian evolution component, so it is not alive. Despite this, a deep understanding of their dynamic is key to improve our knowledge on the subject. A simple (M,R)-system was proposed^12–14^ consisting of three interlinked catalytic cycles, with S, T, and U as external precursors that feed the system, and produce the metabolites ST, SU, and STU, that are degraded during the reaction.

Previous studies found that the system exhibits hysteretic behavior. In the bifurcation diagram, the decay constants act as the bifurcation parameter^15^, separating the parameter space where it can and cannot sustain a self-sustained reaction. The hysteretic behavior may be fundamental, as it allows the system to keep working in the presence of fluctuations beyond a critical point. These attributes were also found by Piedrafita et al.^16^ on a stochastic variant of the (M,R)-system.

Here, we will study the bi-stability of the simple (M,R)-system for the external precursors S, T, and U. It seems more reasonable to approach the bifurcation diagram for the inputs as they regulate the internal dynamics, and because they can be directly controlled. Furthermore, in real systems, which are extended into space by nature, the concentration of the precursors varies at every point. Our first research question is whether such a hysteretic behavior exists in the bifurcation diagram for the external precursors. According to Piedrafita et al.^17^ “All known metabolisms are *vectorial*^18^: they involve gradients, processes occurring in compartmentalized space, diffusion and transport of compounds across diverse boundaries”. Therefore, nutrients do not directly arrive at every point of the protocell, but in general, these molecules can diffuse around the system and produce spatially varying concentrations. Such restrictions, and also the existence of fluctuations, should put certain restraints on the size of the system and its ability to recover from strong perturbations. The external precursors should control these constraints. Therefore, as an illustration, we propose to extend the simple (M, R)-system of Piedrafita et al.^15^ and simulate a one-dimensional extended version that allows diffusion to and within the protocell. Then, the study of the spatially varying external precursors as drivers becomes even more relevant as they move along the protocell with varying concentrations. Here, we plan to study the existence of a critical size in which the system can self-sustain its metabolism, which could mean to be alive. Then, we analyze if such an extension of the system preserves bi-stability and robustness. Finally, we show that two and three-dimensional extensions of the (M,R)-system are viable since a future study of them could be representative of the more realistic spatial distribution of protocells.

The manuscript is organized as follows. In section "The (M,R)-system" we analyze the (M,R)-system and its bi-stable bifurcation diagram. In section "A one-dimensional extended version of the (M,R)-system" we consider the one-dimensional extended (M,R)-system. In section "Viability of two and three-dimensional extended versions of the (M,R)-system" we illustrate the viability of two and three-dimensional extensions. In section "Discussion and conclusions" we present the conclusions.

## The (M,R)-system

Robert Rosen states that only organisms are “close to efficient causation”^19^, as the organism itself produces the catalysts a living organism needs^20^. Although Rosen’s assertion that “a system close to efficient causation cannot have computable models” is controversial^15^, Cornish-Bowden and Letelier et al.^12–14^ provide a concrete and complete example of a simple (M, R)-system comprising three interlinked catalytic cycles:

1 $$\begin{aligned} \text{S} + \text{T}&\xrightarrow {\text{STU}} \text{ST} \ , \end{aligned}$$

2 $$\begin{aligned} \text{ST} + \text{U}&\xrightarrow {\text{SU}} \text{STU} \ , \end{aligned}$$

3 $$\begin{aligned} \text{S} + \text{U}&\xrightarrow {\text{STU}} \text{SU} \ , \end{aligned}$$

where S, T, and U are external precursors. Reaction (1) is a metabolic process that produces a metabolite ST catalyzed by STU, produced by the reaction (2), a replacement process catalyzed by SU. Reaction (3) is a replacement process too, which produces ST catalyzed by STU. The replacement processes are important as ST, SU, and STU are metabolites that have a finite lifetime. Therefore, each one is degraded during the reactions.

**Figure 1:**
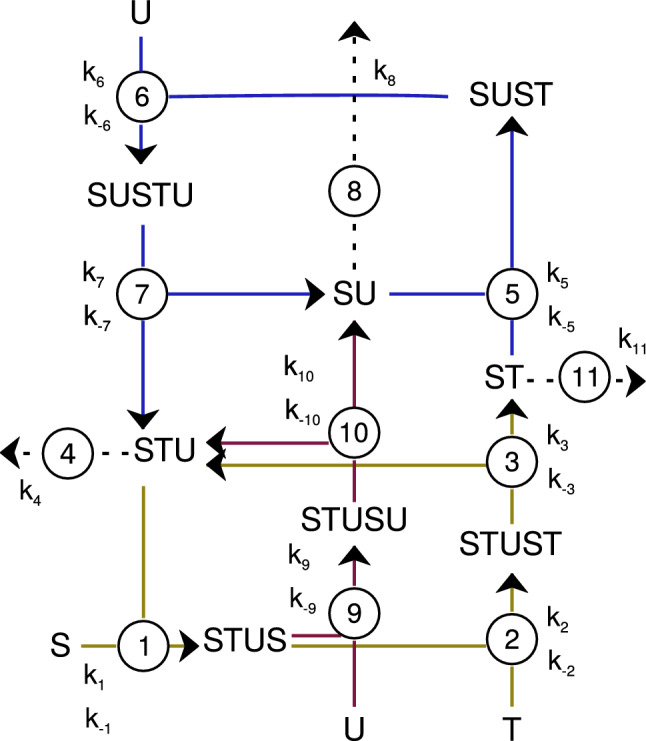
Network diagram of the (M,R)-system based on Piedrafita’s^15^. Each color corresponds to an expanded catalyzed reaction: the metabolic process is in golden yellow, the replacement process is in blue and the replacement of the replacement catalyst is in red. Every reaction in the direction of the arrow was also assigned a rate constant. Steps 4, 8, and 11 denote degradation reactions, and they are assumed to be uncatalyzed and irreversible. The rate constant’s values were taken from Ref.^17^ and they are in Table 1 (section “Methods”).

Piedrafita et al.^15^ were pioneers simulating these cycles extensively. They used differential equations to represent a simple self-maintaining metabolic system in such a way that satisfied three important features for life: robustness, autocatalysis, and bi-stability. Figure 1 shows a network diagram of the (M,R)-system based on Piedrafita’s^15^.

A hysteretic behavior was described by Piedrafita et al.^15^ using the decay constants as parameters of the bifurcation diagram showing the hysteresis cycle. From our physicists’ intuition, we consider those degradation constants as intrinsic parameters of the system. The external precursors seem to be a more natural forcing, or bifurcation parameter, as they can be directly controlled, given fixed decay constants. These allow approaching a fundamental aspect of life as is ‘feeding’. Then, we propose the external precursors’ concentration as drivers of the (M,R)-system. The eight dynamic equations (1)–(8) in the Supplementary material, discarding the diffusion term that we will introduce further in the manuscript, can be written in short-hand notation:

4 $$\begin{aligned} \dot{\mathbf{x}}=\mathbf{F}(\mathbf{x}) , \end{aligned}$$

where $\mathbf{x}=\left( [\text{STU}],[\text{ST}],[\text{STUS}],[\text{STUST}],[\text{ST}],[\text{SUST}],[\text{SUSTU}],[\text{STUSU}]\right)$ is a vector with the concentrations of the metabolites, the dynamical variables of the system, and **F** is a nonlinear vector field that depends on the vector $\mathbf {x}(t)$. This vector field also depends on the external precursors (S, T, and U) and the constant rates $k_i$ that are described in Table 1. Here we can find stable and unstable equilibrium solutions by solving

5 $$\begin{aligned} 0=\mathbf{F}(\mathbf{x}^{\star }) . \end{aligned}$$

**Figure 2:**
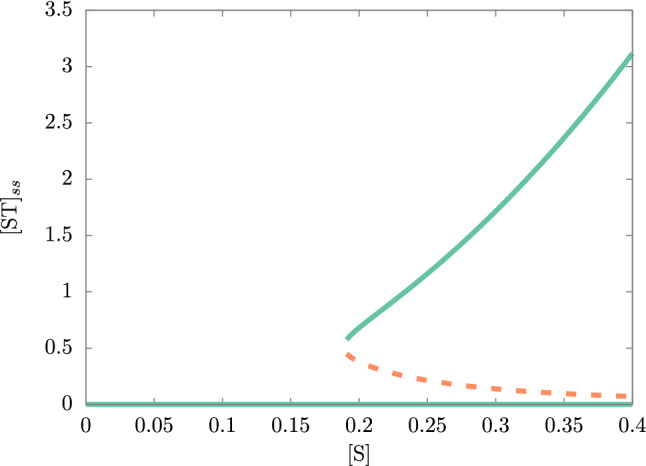
Bifurcation plot of the (M,R)-system’s $[\text{ST}]_{ss}$ with $[\text{S}]=[\text{T}]=[\text{U}]$ as bifurcation parameters. The stable branch is in green and the unstable one is in orange. The critical value, where the stable and unstable branch split-up, is $[\text{S}]=0.19$. The rate constant’s values were taken from Ref.^17^ and they are in Table 1 (section “Methods”).

The stability of the steady-state solutions can be ascertained from the eigenvalues of the corresponding Jacobian matrix evaluated on the steady-state solution $\mathbf{DF}(\mathbf{x}^{\star })$. If all eigenvalues have a negative real part then the equilibrium is stable, otherwise; it is unstable. Also, we must discard solutions for $\mathbf{x}^{\star }$ with negative entries as they lack physical meaning. In Fig. 2, a bifurcation plot is shown for the relevant stable (continuous lines) and unstable (dashed line) equilibrium solutions as we vary $[\text{S}]=[\text{T}]=[\text{U}]$.

For concentrations of the external precursors below the critical value $[\text{S}] = 0.19$, the system undoubtedly cannot be functional, since a fundamental requirement to this is the self-sustainability of the chemical system, and in this trivial stable branch we found a zero concentration of metabolites as shown for $[\text{ST}]_{ss} = 0$. Colloquially, we could say that the system is “dead”. However, as the concentration $[\text{S}] = [\text{T}] = [\text{U}]$ goes beyond that critical value, we find a region where two stable steady-states coexist, the trivial or death state and the non-trivial one. An unstable steady-state separates both stable steady-states. This defines a hysteretic loop. Note that the bi-stability determines whether the system can or cannot be self-sustainable (“alive”), understanding the non-trivial stable steady-state as the possibility of finding a self-sustaining chemical system, which means non-zero concentrations for the metabolites as it does happen for $[\text{ST}]_{ss}$ in the Fig. 2.

## A one-dimensional extended version of the (M,R)-system

Piedrafita et al.^21^ has suggested that nutrients accessibility becomes relevant when dealing with protocells. Thus, we simulate a protocell with spatially varying concentrations. The concentrations will now vary in one dimension (1D) with a defined size, so at every point across this 1D space, we have an (M,R)-system. For simplicity, the nutrient transport process along the protocell is modeled by a diffusive transport^22^. All the molecules move from points of greater to lower concentration, a more realistic representation of a protocell as the molecules are not found homogeneously within it. In essence, diffusion aims to balance concentrations between adjacent points in the protocell, so, the more different the concentrations, the faster the transfer of metabolites. Then, the differential equations given by Piedrafita et al.^15^ change by adding a new term that accounts for the diffusion of the form

6 $$\begin{aligned} D=D_{c}\dfrac{\partial ^{2}[c]}{\partial x^{2}}, \end{aligned}$$

where *c* indicates a particular metabolite, as we show in Eqs. (1)–(8) in the Supplementary material. To allow the variation of precursors’ concentration inside the protocell, we add three extra equations to the (M, R)-system, that ensure the conservation of quantity of each element. These extra equations, (9) to (11) in the Supplementary material, are necessary because the system is fed with external precursors only on the boundaries, namely, at $x=0$ and $x=L$. We also assume, like Ref.^17^, that there is an external environment rich on precursors (S, T, and U), which are practically unlimited (primordial-soup hypothesis). We represent this mathematically by setting $[\text{S}](x=0, t)$, $[\text{T}](x=0, t)$, $[\text{U}](x=0, t)$, $[\text{S}](x=L, t)$, $[\text{T}](x=L, t)$, and $[\text{U}](x=L, t)$ to a constant value. Among the same lines, we assume a semi-permeable membrane that allows only the transport of these small precursors^23,24^, consequently, we set $\partial [c]/\partial x = 0$ for all metabolites but the precursors. Note that the protocell regulates by itself the input diffusion of external precursors, unlike Ref.^17^. Also, note that the simulation is symmetric respect to $x = L/2$ if we set the concentration of precursors to the same value in both boundaries. For simplicity, we will address the case $[\text{S}](x=0, t)=[\text{T}](x=0, t)=[\text{U}](x=0, t) = [\text{S}](x=L, t) = [\text{T}](x=L, t) = [\text{U}](x=L, t)$. It is important to realize that if the diffusion constant is null, then the protocell cannot self-maintain its metabolism since it cannot interact with the environment to “feed” itself with the external precursors. Using the extended version of the equations, we simulate the 1D (M, R)-system.

**Figure 3:**
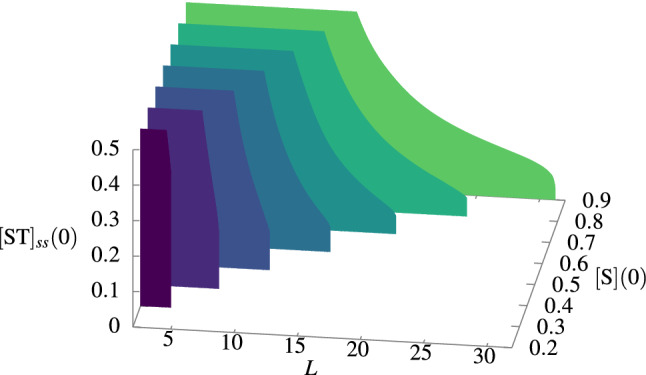
Three-dimensional plot of $[\text{ST}]_{ss}(0)$ for several concentrations of the external precursors $[\text{S}](0)=[\text{T}](0)=[\text{U}](0)$ and sizes of the protocell. Each color denotes a two-dimensional plot of $[\text{ST}]_{ss}(0)$ as a function of *L*. Here, the diffusion constant value is $D_{c}=1$. The rate constant’s values were taken from Ref.^17^ and they are in Table 1 (section “Methods”).

It has been suggested that the system’s volume may have been a restriction to the first proto-metabolic networks’ maintenance. Furthermore, that this physical requirement will affect other theories of living organizations assuming confinement in a physical compartment^16^. So, let’s suppose that a permanent source of the external precursors is located at both ends of the 1D protocell as a constant reservoir. The rate of the external precursors determines if the entire protocell can or cannot be self-sustainable ( “alive” or “dead”). This rate depends on a balance between the size of the system and the diffusion coefficients that preserves metabolism. In Fig. 3, we show the asymptotic value of the concentration $[\text{ST}](x=0)$ for several concentrations $[\text{S}](x=0)=[\text{T}](x=0)=[\text{U}](x=0)$ and size *L* that can be supported. Beyond the maximum value of *L*, there is no system as the concentration of the metabolites goes to zero.

**Figure 4:**
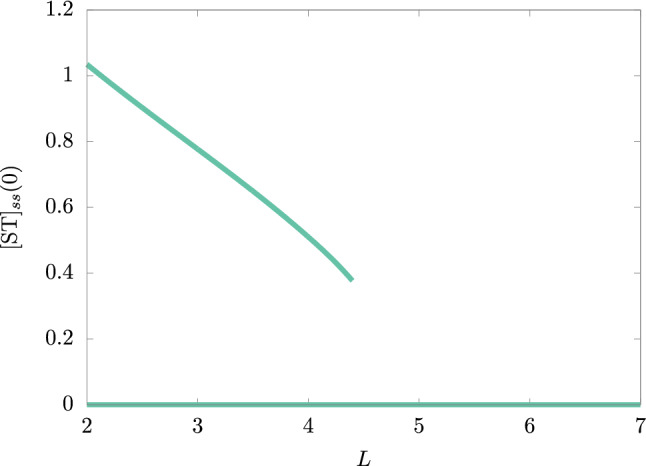
Bifurcation plot of the extended (M,R)-system’s $[\text{ST}]_{ss}(0)$ with the protocell size *L* as the bifurcation parameter. Here, the diffusion constant value is $D_c=1$. The rate constant’s values were taken from Ref.^17^ and they are in Table 1 (section “Methods”).

Given a fixed value of *L*, as $[\text{S}](0)$ increases, $[\text{ST}](0)$ also increases. The hysteretic behavior is preserved as it is similar to the bifurcation diagram of Fig. 2. This means that lower concentrations of the external precursors at the feeding point $x=0$ make it impossible to keep the production of ST at a sufficient level to feed the protocell. Hence, for a fixed value of $[\text{ST}](0)$, we note that there is an equivalent bifurcation diagram, such that if we take $[\text{ST}](0)=0$ we see that the bigger the value of *L*, the greater the required concentration $[\text{S}](0)$ (and the other precursors) to keep the condition of self-sustaining of the chemical system. Similarly, for a fixed value of $[\text{S}](0)$, we found that as the size increases, $[\text{ST}](0)$ decreases until the protocell self-sustainability cannot be possible, as shown in Fig. 4 for $[\text{S}](0)=0.3$.

Cornish-Bowden and Piedrafita et al.^25^ found a critical volume for a particular numerical value of concentrations of external precursors. In their words: “Below this volume, the system could not maintain itself for any significant time”. They suggest that there is a minimum size for an (M,R)-system based on stochastic simulations of simple (M,R)-system where statistical fluctuations have important consequences for the system’s dynamics. Here, we found a maximum size such that the protocell can preserve its metabolism, and hence keep functioning, under a fixed condition of the external precursors.

**Figure 5:**
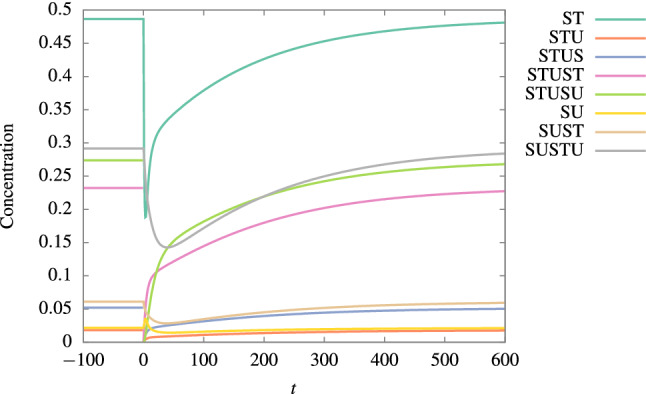
Dynamics of the ongoing recovery from a catastrophic loss of STU, STUS, STUST, and STUSU at time $t=0$. Here, $[\text{S}]=0.6$, $L=5$ and $D_{c}=5$. The rate constant’s values were taken from Ref.^17^ and they are in section “Methods” (Table 1).

Furthermore, we found that this extension of the (M,R)-system preserves the robustness observed in Ref.^15^. Likewise the system can recover its metabolism from an abrupt loss of most of its molecules once it is in the stable non-trivial steady state of concentrations. To test such an effect in the extended system, in Fig. 5 we show the system’s dynamics subject to a catastrophic loss of STU, STUS, STUST, and STUSU at time $t=0$. We observe the ongoing recovery of the initial concentrations in all the molecules. Hence, the notion of robustness is preserved in these protocells.

## Viability of two and three-dimensional extended versions of the (M,R)-system

As far as we know, life forms unfold in two and three dimensions. The question is whether a 2D and 3D extended versions of the (M,R)-system can be simulated, while maintaining the diffusion model previously studied in section "A one-dimensional extended version of the (M,R)-system". To do so, we change the diffusion term so that now the protocell is extended into a polar and spherically symmetric space, respectively:

7 $$\begin{aligned} D_{cyl}&=D_{c}\dfrac{1}{r}\dfrac{\partial }{\partial r}\left( r\dfrac{\partial [c]}{\partial r}\right) \,, \end{aligned}$$

8 $$\begin{aligned} D_{sph}&=D_{c}\dfrac{1}{r^{2}}\dfrac{\partial }{\partial r}\left( r^{2}\dfrac{\partial [c]}{\partial r}\right) \,. \end{aligned}$$

**Figure 6:**
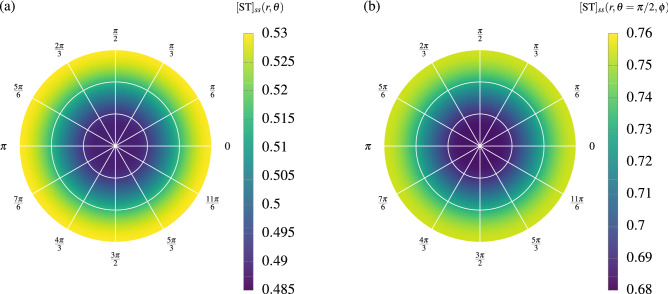
Cross-section of two different shapes of protocell, where the color shows the value of $[\text{ST}]_{ss}$ at every point. Symmetric protocells in (**a**) two and (**b**) three dimensions. Both simulations were done using the same parameters, namely: size $R=0.5$, reservoir of external precursors $[\text{S}]=0.5$, and the diffusion constant $D_c=1$. The rate constant’s values were taken from Ref.^17^ and they are in Table 1 (section “Methods”).

Analogously to the previous section, the protocell is fed by a permanent source of the external precursors at the maximum radius. Note that for the 2D protocell the edge is a perimeter, and the 3D protocell is a surface. Here, we also set to zero the decay constants and the spatial derivative of the metabolites to zero at the edge of the cell. In Fig. 6, we show a cross-section of these polar and spherically symmetric protocells, where the color shows the value of $[\text{ST}]_{ss}$ at every point.

As we expected, metabolites diffuse from the maximum radius $R=0.5$ to the center of the protocell, in both cases. Consequently, the concentration of the metabolites increases in the radial direction, as we have shown for $[\text{ST}]_{ss}$. Note that the 3D case reaches higher concentrations of ST, which is a consequence of the ratio between hyper-volume of the protocell (the one to feed) and the hyper-area that enclose it (who feed it) decreases with the dimension; i.e. *R*/2 for two dimensions and *R*/3 for three dimensions, respectively. In the general case, this fraction goes as *R*/*d*.

Hence, we would expect that a larger symmetric structure can be formed in three dimensions. One way to allow for an even larger structure is to increase the surface area, for example, by having a fractal boundary. The two-dimensional case is still interesting, since autocatalysis is often related to surface reactions.

## Discussion and conclusions

*Metabolism-replacement systems* provide a straightforward example of processes that contain many of the must-have ingredients for life. Previous works found a bi-stable behavior of the system using the decay constants as the system’s control parameters, simulated in a stochastic and non-stochastic manner^15,16^. We propose that the natural external control parameters of the system be the regulators of internal dynamics, namely the external precursors. Hence, we found that using the external precursors as drivers, the bi-stable feature is preserved so there is a hypervolume of initial conditions for the external precursors where self-sustainability can be possible.

Using a diffusion model, we simulated a 1D protocell made of (M, R)-systems. We found that when the system is fed by a permanent source of external precursors at both of its ends, the bi-stable behavior is also preserved when using the external precursors as drivers. Also, we observed that a critical size exists, which limits the viability of self-sustaining its metabolism. So, it restricts the region where functionality is possible, for fixed values of the external precursors.

Leyva et al.^22^ concluded that “cell size should be a key factor determining the potential of these primitive systems to evolve and consequently to support life”. As we mentioned before, Cornish-Bowden and Piedrafita et al.^21^ suggested the existence of a minimum size for an (M,R)-system. We proposed that a maximum size should limit nutrients’ accessibility along a 1D protocell. Moreover, we demonstrated that this extension of the system is robust, so this one-dimensional extended version of the (M,R) system preserves robustness, autocatalysis, and bi-stability as suggested by Piedrafita et al.^15^.

Finally, we show that it is also feasible to extend the system to two and three dimensions, at least in the polar and spherically symmetric cases, and found that dimension and shape affect the diffusion of precursors within the protocell. We hope to thoroughly analyze this case, with all the complexity that this brings, in our next work. For example, we may consider the inclusion of stochasticity is inherent in the reactions of these systems, as discussed in Ref.^16^ for the simplified (M,R)-system model without diffusion. Such effects are expected to generate more complex surface and volumetric structures. Another question that remains open is whether exists a spatial configuration that optimizes feeding and auto sustainability, which suggests looking into fractal boundaries.

## Methods

All simulations were done using the rate constant’s values of Reference^17^, namely.

**Table 1:** Rate constant’s values used in all simulations.

First-order reactions [$\tau ^{-1}$]	Second-order reactions [$\text{mM}^{-1}\,\tau ^{-1}$]
$k_{-1}=10$	$k_{1}=100$
$k_{-2}=10$	$k_{2}=100$
$k_{3}=2$	$k_{-3}=10$
$k_{-5}=1$	$k_{5}=10$
$k_{-6}=1$	$k_{6}=10$
$k_{7}=0.1$	$k_{-7}=1$
$k_{-9}=0.05$	$k_{9}=1$
$k_{10}=0.05$	$k_{-10}=0.5$
$$k_{4}=0.3$$	
$$k_{8}=0.3$$	
$$k_{11}=0.3$$	

These were extracted from Reference^17^.

## Supplementary information

Supplementary material
