# Supplementary material for: Stability and robustness of asymptotic autocatalytic systems
Source: Sci Rep. 2020 Sep 23;10:15498. doi: 10.1038/s41598-020-72580-9 (PMC7511346; doi:10.1038/s41598-020-72580-9)
Supplement: Supplementary file 1 — Supplementary material [file 41598_2020_72580_MOESM1_ESM.pdf]

Supplementary information to:

Stability and robustness of asymptotic autocatalytic systems

Sohyoun Yun-Cárcamo, Sebastián Carrasco, José Rogan, Paulina Correa-Burrows, Juan Alejandro Valdivia

The equations of the model with the diffusion term are given by

$$\frac{\partial[\mathrm{STU}]}{\partial t} = -k_1[\mathrm{STU}][\mathrm{S}] + k_{-1}[\mathrm{STUS}] + k_3[\mathrm{STUST}] - k_{-3}[\mathrm{STU}][\mathrm{ST}] - k_4[\mathrm{STU}] + k_7[\mathrm{SUSTU}] - k_{-7}[\mathrm{STU}][\mathrm{SU}] + k_{10}[\mathrm{STUSU}] - k_{-10}[\mathrm{STU}][\mathrm{SU}] + D_{STU}\frac{\partial^2[\mathrm{STU}]}{\partial x^2}, \quad (1)$$

$$\frac{\partial[\mathrm{SU}]}{\partial t} = k_7[\mathrm{SUSTU}] - k_{-7}[\mathrm{STU}][\mathrm{SU}] - k_5[\mathrm{ST}][\mathrm{SU}] + k_{-5}[\mathrm{SUST}] - k_8[\mathrm{SU}] + k_{10}[\mathrm{STUSU}] - k_{-10}[\mathrm{STU}][\mathrm{SU}] + D_{SU}\frac{\partial^2[\mathrm{SU}]}{\partial x^2}, \quad (2)$$

$$\frac{\partial[\mathrm{STUS}]}{\partial t} = k_1[\mathrm{STU}][\mathrm{S}] - k_{-1}[\mathrm{STUS}] - k_2[\mathrm{STUS}][\mathrm{T}] + k_{-2}[\mathrm{STUST}] - k_9[\mathrm{STUS}][\mathrm{U}] + k_{-9}[\mathrm{STUSU}] + D_{STUS}\frac{\partial^2[\mathrm{STUS}]}{\partial x^2}, \quad (3)$$

$$\frac{\partial[\mathrm{STUST}]}{\partial t} = k_2[\mathrm{STUS}][\mathrm{T}] - k_{-2}[\mathrm{STUST}] - k_3[\mathrm{STUST}] + k_{-3}[\mathrm{STU}][\mathrm{ST}] + D_{STUST}\frac{\partial^2[\mathrm{STUST}]}{\partial x^2}, \quad (4)$$

$$\frac{\partial[\mathrm{ST}]}{\partial t} = k_3[\mathrm{STUST}] - k_{-3}[\mathrm{STU}][\mathrm{ST}] - k_5[\mathrm{ST}][\mathrm{SU}] + k_{-5}[\mathrm{SUST}] - k_{11}[\mathrm{ST}] + D_{ST}\frac{\partial^2[\mathrm{ST}]}{\partial x^2}, \quad (5)$$

$$\frac{\partial[\mathrm{SUST}]}{\partial t} = k_5[\mathrm{ST}][\mathrm{SU}] - k_{-5}[\mathrm{SUST}] - k_6[\mathrm{SUST}][\mathrm{U}] + k_{-6}[\mathrm{SUSTU}] + D_{SUST}\frac{\partial^2[\mathrm{SUST}]}{\partial x^2}, \quad (6)$$

$$\frac{\partial[\mathrm{SUSTU}]}{\partial t} = k_6[\mathrm{SUST}][\mathrm{U}] - k_{-6}[\mathrm{SUSTU}] - k_7[\mathrm{SUSTU}] + k_{-7}[\mathrm{STU}][\mathrm{SU}] + D_{SUSTU}\frac{\partial^2[\mathrm{SUSTU}]}{\partial x^2}, \quad (7)$$

$$\frac{\partial[\mathrm{STUSU}]}{\partial t} = k_9[\mathrm{STUS}][\mathrm{U}] - k_{-9}[\mathrm{STUSU}] - k_{10}[\mathrm{STUSU}] + k_{-10}[\mathrm{STU}][\mathrm{SU}] + D_{STUSU}\frac{\partial^2[\mathrm{STUSU}]}{\partial x^2}. \quad (8)$$

We also add the following equations to the original model, as we let the precursors to vary inside the protocell.

$$\frac{\partial[\mathrm{S}]}{\partial t} = -k_1[\mathrm{STU}][\mathrm{S}] + k_{-1}[\mathrm{STUS}] + D_S\frac{d^2[\mathrm{S}]}{\partial x^2}, \quad (9)$$

$$\frac{\partial[\mathrm{T}]}{\partial t} = -k_2[\mathrm{STUS}][\mathrm{T}] + k_{-2}[\mathrm{STUST}] + D_T\frac{\partial^2[\mathrm{T}]}{\partial x^2}, \quad (10)$$

$$\frac{\partial[\mathrm{U}]}{\partial t} = -k_6[\mathrm{SUST}][\mathrm{U}] + k_{-6}[\mathrm{SUSTU}] - k_9[\mathrm{STUS}][\mathrm{U}] + k_{-9}[\mathrm{STUSU}] + D_U\frac{\partial^2[\mathrm{U}]}{\partial x^2}. \quad (11)$$

Note that these equation ensure the conservation of the quantity of each element, except for the degradation process of ST, SU and STU.
